# *In Situ* Stability of Anthocyanins in *Lycium ruthenicum* Murray

**DOI:** 10.3390/molecules26237073

**Published:** 2021-11-23

**Authors:** Yanping Wang, Jingxian Fu, Dong Yang

**Affiliations:** 1 Beijing Key Laboratory of Functional Food from Plant Resources, College of Food Science & Nutritional Engineering, China Agricultural University, 17 East Tsinghua Rd., Beijing 100083, China; wyp432@163.com (Y.W.); 2017306100215@cau.edu.cn (J.F.); 2 Department of Biological Engineering, Vocational Technology College of Bayin Guoleng, Korla 841000, China

**Keywords:** anthocyanin, *Lycium ruthenicum* Murray, stability

## Abstract

In this research, the effects of drying method, storage temperature, and color protector glucose on anthocyanin preservation in the *Lycium ruthenicum* Murr. fruit were studied. Compared with hot-air drying, vacuum freeze-drying preserved about 5.8-fold more anthocyanins. The half-life of anthocyanins in the freeze-dried fruit samples with glucose was 3.6 days, 1.8 days, and 1.7 days at 4 °C, 20 °C, and 37 °C, respectively. On the other hand, the half-life values without glucose addition were 2.2 days, 2.3 days, and 2.1 days at each temperature, respectively, indicating that glucose protected anthocyanins at low temperature. The composition and contents of anthocyanins and anthocyanidins in the freeze-dried *Lycium ruthenicum* Murr., stored for 20 days, were investigated with a HPLC-MS/MS setup. It was found that most anthocyanidins in *Lycium ruthenicum* Murr. are linked with coumaroyl glucose to form anthocyanins, while glycosylated and acetyl-glycosylated anthocyanins were also detected. Five anthocyanidins were detected: delphinidin, cyanidin, petunidin, malvidin, and peonidin, and delphinidin accounts for about half of the total amount of anthocyanidins. It is much more economic to conserve anthocyanins *in situ* with freeze-drying methods and to store the fruits at low temperatures with glucose.

## 1. Introduction

*Lycium ruthenicum* Murray, also called black goji, is a Chinese herb widely distributed in the northwestern part of China including Xinjiang, Tibet, Qinghai, and Gansu provinces [1,2]. It has exhibited multiple beneficial effects: alleviating nonalcoholic fatty liver, protecting cortical neurons and ameliorating Alzheimer’s disease, memory impairment, oxidative stress, and neuroinflammation, protective effects against radiation injury, immunomodulating activity, inhibiting pancreatic cancer cell growth and antiproliferative effects, antioxidant activity against isoproterenol-induced acute myocardial ischemia, promoting osteoblast differentiation, and reducing nicotine withdrawal-induced anxiety [3,4,5,6,7,8,9,10,11,12,13]. The majority of these beneficial health effects are related to anthocyanins that endow the black–blue color to the fruit [14]. Thus, lots of studies have been focusing on anthocyanin extraction methods from *Lycium ruthenicum* Murr. [15,16,17,18].

Meanwhile, numerous efforts have been devoted to enhancing the stability of anthocyanins in *Lycium ruthenicum* Murr., mostly in the solution form [19,20]. However, little attention is paid to the stability and preservation of anthocyanins *in situ* (in the fruit). The stability of anthocyanins while they are still in the fruit matters since there is often a long time before they get further processed into a variety of products, not to mention that *Lycium ruthenicum* Murr. is often consumed in its fruit form where the anthocyanin content varies during processing [21]. Here, in this study, we firstly studied the effect of different drying methods and storage conditions on the stability of anthocyanins *in situ* in *Lycium ruthenicum* Murr. and analyzed the composition and content of anthocyanins in freeze-dried *Lycium ruthenicum* Murr. fruit after a certain storage period. This study highlights the great loss of anthocyanins during improper processing and attempts to find a more economic way to preserve them.

## 2. Results

### 2.1. Effect of Different Drying Methods on the Stability of Anthocyanins in Lycium ruthenicum Murr.

**Figure 1:**
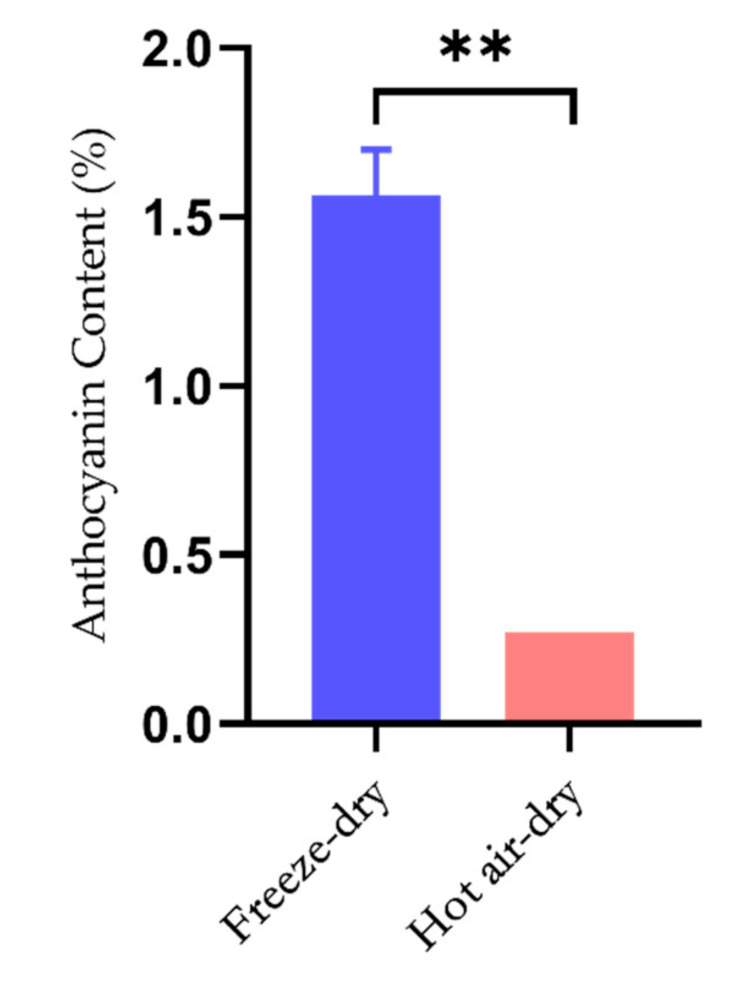
Effect of different drying methods on the preservation of anthocyanins in *Lycium ruthenicum* Murr. Freeze-dried *Lycium ruthenicum* Murr. contains much higher content of anthocyanin than hot air-dried ones. ** indicates that *p* < 0.01.

As shown in Figure 1, the total anthocyanin content in *Lycium ruthenicum* Murr. reached about 1.56% once the fruits were completely dried by the freeze-drying method. On the other hand, the total anthocyanin content decreased to about 0.27% after the fruits were completely dried via the hot-air drying method. The anthocyanin content is significantly higher in freeze-dried fruit (about 5.8 fold) than that in the hot-air dried fruits (*p* < 0.01).

### 2.2. Effect of Temprature and Glucose on the Stability of Anthocyanins in Lycium ruthenicum Murr.

**Figure 2:**
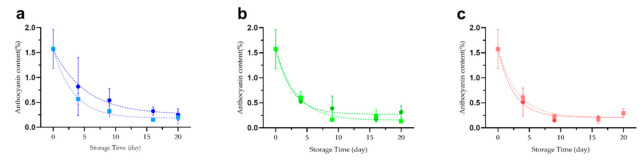
Effect of temperature and glucose on the preservation of anthocyanins in *Lycium ruthenicum* Murr. The anthocyanin content of freeze-dried *Lycium ruthenicum* Murr. at (**a**) 4 °C, (**b**) 20 °C, (**c**) 37 °C in the presence (filled circle) and absence (filled square) of glucose.

Glucose was added to the freeze-dried *Lycium ruthenicum* Murr., and samples with and without glucose addition were stored at different temperatures to examine the storage conditions on the preservation of anthocyanins. The half-life of anthocyanins in the presence of glucose is about 3.6 days at 4 °C, while that in the absence of glucose is about 2.2 days (Figure 2a, Table 1). Once the temperature was increased to 20 °C, the half-life of anthocyanins in the fruit decreased to 1.8 days in the presence of glucose while it remained at about 2.3 days in the absence of glucose (Figure 2b, Table 1). As the temperature continued to increase to 37 °C, the half-life of *in situ* anthocyanins further decreased to about 1.7 days in the presence of glucose while it remained at about 2.1 days in the absence of glucose (Figure 2c, Table 1).

Once the data were reexamined, it was apparent that the half-life values of *in situ* anthocyanins in the fruits were 2.2, 2.3, and 2.1 days at 4 °C, 20 °C, and 37 °C, respectively. Once glucose was added, the half-life at 4 °C increased to 3.6 days, much longer than that at 20 °C and 37 °C.

### 2.3. Composition Analysis and Quantification of Anthocyanins in Freeze-Dried Lycium ruthenicum Murr.

**Figure 3:**
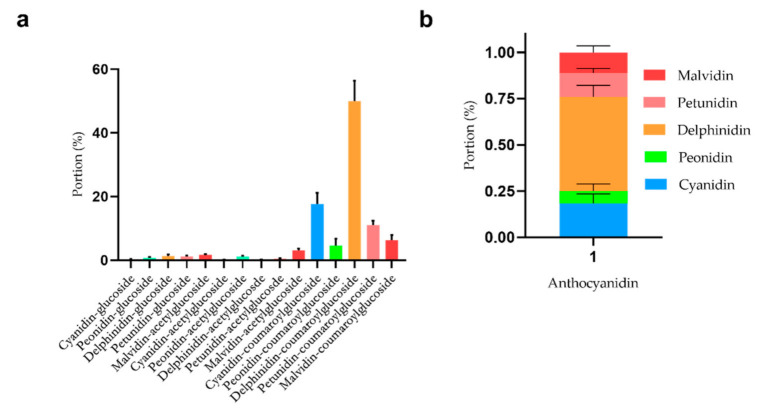
Composition analysis and quantification of anthocyanins in *Lycium ruthenicum* Murr. The portion of anthocyanins (**a**) and anthocyanidins (**b**) in freeze-dried *Lycium ruthenicum* Murr.

The freeze-dried *Lycium ruthenicum* Murr. was stored for 20 days before composition and content analysis by the HPLC-MS/MS setup. It can be concluded that the major composition is the coumaroylglucoside-conjugated anthocyanidins in the freeze-dried fruits (Figure 3a). Among them, delphinidin-coumaroylglucoside accounts for about 42–56%, cyanidin-coumaroylglucoside for about 13–21%, petunidin-coumaroylglucoside for about 9–12%, and malvidin-coumaroylglucoside for about 5–8%.

The anthocyanidins listed from high to low in terms of their relative content are delphinidin, cyanidin, petunidin, malvidin, and peonidin, respectively. Among them, delphinidin is the major anthocyanin that accounts for about 51% of the total anthocyanidins *in situ*.

## 3. Discussion

Drying lowers the water content in the fruits. As such, it slows down the degradation of the fruits, including anthocyanins *in situ*, and lowers the transportation expense. The hot-air drying method is the least expensive method that requires few facilities, thus it is widely used in northwestern China to dehydrate agro-products. Most of the *Lycium ruthenicum* Murr. samples are dried locally by hot air without additional treatment. Our data indicate that this method greatly compromised the anthocyanin content *in situ*, which is the major attribute of this fruit. Thus, it is more economic to persevere the anthocyanins in the fruits with the freeze-dry method.

The addition of glucose was supposed to protect the anthocyanins in purple potato peel *in situ* and anthocyanins in the blueberry juice in solution, and it is reported that copigmentation of glucose at different ratios did not affect the stability of anthocyanin [22,23]. In this study, the addition of glucose in the freeze-dried *Lycium ruthenicum* Murr. prolonged the half-life at 4 °C, but not at 20 °C, or 37 °C. The deprotonation of the anthocyanin flavylium ion to form quinonoid base and hydration to form hemiketal were proven to be endothermic [24]. Thus, elevating the storage temperature brought about more rapid degradation of anthocyanins. However, once glucose was added to the freeze-dried *Lycium ruthenicum* Murr. at low temperature (4 °C in this study), the anthocyanins were protected *in situ*. It is highly likely that the anthocyanin stability in the fruits was enhanced by the copigmentation with glucose, possibly via glycosylation with glucose [22]. Thus, it is only useful to add glucose to protect the anthocyanins when the fruits are stored at low temperature ~4 °C.

Our anthocyanin composition analysis is basically consistent with previous publications, except that we also found peonidin and cyanidin in the anthocyanidin composition, and their glucoside and acetyl glucoside derivatives [25]. This is possibly because the freeze-drying method better protected these rare species that allowed us to detect them, and/or due to a slight difference between different *Lycium ruthenicum* Murr. samples.

## 4. Materials and Methods

### 4.1. Materials

Fresh *Lycium ruthenicum* Murr. whole fruits were collected from Kuerle, Xinjiang Province in September 2020. Ethanol was purchased from Modern Oriental Fine Chemistry (Beijing, China). Sodium acetate of analytical grade was purchased from XiLong Scientific (Shantou, Guangdong Province, China). Hydrochloric acid of analytical grade, methanol, and formic acid of HPLC grade were purchased from Sinopharm Chemical Reagent Co., Ltd. (Beijing, China).

### 4.2. Methods

#### 4.2.1. Drying Methods

*Lycium ruthenicum* Murr. fruits were hot-air dried on a glass Petri dish in an oven (Shanghai Yiheng Scientific Instruments, Shanghai, China) with the temperature set at 50 °C until the weight change of the fruits was less than 0.01 g. Alternatively, *Lycium ruthenicum* Murr. fruits were freeze dried in an LGJ-10 vacuum freeze dryer (Beijing Huaxing Technology Development Co., Ltd. Songyuan, Beijing, China) at 10 pa until the weight was stable.

#### 4.2.2. Storage Conditions

Freeze-dried *Lycium ruthenicum* Murr. fruits were placed in an Erlenmeyer flask under atmospheric packaging sealed with parafilm, with each flask containing about 40 g of the fruits. Half of the samples were mixed with 2 g glucose powder in each flask, and stored at 4 °C, 20 °C, and 37 °C in parallel with the other half of the samples with no glucose addition. The anthocyanin content was measured at different time intervals as described in the following.

#### 4.2.3. Anthocyanin Content Determination

*Lycium ruthenicum* Murr. fruits of 0.2–0.3 g were grinded, mixed with 80% ethanol, and extracted in an ultrasonic water bath (GT Sonic, Meizhou, Guangdong Province) for 30 min at 35 °C. The extracted anthocyanin content was measured by adding 20 μL of the supernatant to 980 μL of hydrochloric acid solution pH 1.0, or sodium acetic buffer pH 4.5. The levels of absorbance at 526 nm and 700 nm of these two solutions were measured with a UV–Vis spectrophotometer (UV-2450, Shimadzu, Kyoto, Japan). The anthocyanin content was calculated according to previous studies [26,27].

#### 4.2.4. Anthocyanin Composition and Quantification

The composition and content of each anthocyanin was determined using HPLC-DAD-MS/MS with a 1290 Series liquid chromatography system (Agilent Technologies Inc., Palo Alto, CA, USA) as previously published [28,29,30].

#### 4.2.5. Data Analysis

The degradation kinetic experiment was performed independently 3 times, and data were fitted with the one-phase decay equation. The anthocyanin contents experiment was performed independently 4 times. Statistical analysis was performed with the t-test, and graphs were all made the with Prism software (v. 8.0, GraphPad Software Inc., La Jolla, CA, USA).

## 5. Conclusions

Compared with the commonly used hot air-drying method, the freeze-dry method better preserved the anthocyanins in *Lycium ruthenicum* Murr. Glucose prolonged the half-life of anthocyanins *in situ* at low temperatures, probably due to the copigmentation via glycosylation. Freeze-dried *Lycium ruthenicum* Murr. contains substantial delphinidin, cyanidin, petunidin and their coumaroyl-glucoside derivatives. It is much more economic to protect anthocyanins *in situ* with the freeze-dry method and store the fruits at low temperatures with glucose.

## Figures and Tables

**Table 1:** Half-life of anthocyanin degradation at different storage conditions.

Temp	Half-Life (Day)	
	No Addition	Glucose Addition
4 °C	2.246	3.596
20 °C	2.263	1.801
37 °C	2.123	1.734

## Data Availability

Data are contained within the article.
